# Comparative evaluation of the effect of two pulpal medicaments on pain and bleeding status of mandibular molars with irreversible pulpitis post-failure of inferior alveolar nerve block: a double-blind, randomized, clinical trial

**DOI:** 10.7717/peerj.13397

**Published:** 2022-05-13

**Authors:** Naomi Ranjan Singh, Lora Mishra, Ajinkya M. Pawar, Nike Kurniawati, Dian Agustin Wahjuningrum

**Affiliations:** 1 Department of Conservative Dentistry and Endoodntics, Institute of Dental Sciences, Bhubaneswar, Odisha, India; 2 Department of Conservative Dentistry and Endoodntics, Nair Hospital Dental College, Mumbai, Maharashtra, India; 3 Department of Conservative Dentistry, Faculty of Dental Medicine, Universitas Airlingga, Surabaya City, East Java, Indonesia

**Keywords:** Hyperalgesia, Inferior alveolar nerve block, Irreversible pulpitis, Paraformaldehyde, Visual analogue scale

## Abstract

**Background:**

Complete relief of pain due to irreversible pulpitis is challenging to obtain with analgesic medications. The high incidence of an inferior alveolar nerve block (IANB) failure makes it difficult for practitioners to perform endodontic treatment without implementing other anesthetic techniques, especially mandibular molars. The aim of this study was to compare efficacies of two different quantities of paraformaldehyde based pulpal medicaments to relieve the pain and control hyperemic pulp post-failure of IANB and supplementary technique in patients experiencing this symptomatic irreversible pulpitis in the permanent mandibular tooth.

**Method:**

Eighty-two participants with severe pain pre-operatively (Heft Parker Visual Analogue Scale, VAS > 114 mm) were enrolled, and pain responses were recorded at different time intervals using the Heft Parker visual analogue scale. To the patients experiencing pain even after the administration of the standard IANB and supplemental intraligamentary injection, one of the two paraformaldehyde based pulpal medicaments was placed in the pulp chamber and sealed. Participants were recalled after 24–48 h (second visit) to assess pain and bleeding reduction.

**Results:**

Results showed a significant decrease in pain severity and bleeding score post medicament placement (*p* < .05). Hence judicious use within a recommended period, pulpal medicaments can be considered safe.

**Conclusion:**

Paraformaldehyde based pulpal medicament can be used as an alternative to manage pain in patients having severe irreversible pulpitis and hyperalgesia.

## Introduction

Adequate local anesthesia (LA) is crucial in dental treatment, especially in managing irreversible pulpitis. Posterior teeth are routinely anesthetized in the mandibular arch using the inferior alveolar nerve block (IANB) technique. Unfortunately, IANB is proven to have substantial failure rates ranging between 43%–83% (AlHindi, Rashed & AlOtaibi, 2016; Fowler et al., 2016; Madan, Madan & Madan, 2002; Nagendrababu et al., 2019; Sivaramakrishnan, Alsobaiei & Sridharan, 2019). The reasons could be that patients with symptomatic irreversible pulpitis or acute apical periodontitis are often more apprehensive, which lowers the pain threshold. Moreover, nociceptors exhibit decreased excitability threshold due to chemokines and cytokines released in inflammation (Modaresi, Dianat & Soluti, 2008). The other reason could be that local anaesthetic action reduces the presence of a tetrodotoxin-resistant class of sodium channels (Roy & Narahashi, 1992). Additionally, there is a relatively high inaccuracy of IANB block due to needle deflection (Kennedy et al., 2003; Khalil, 2014).

These failures may be overcome by administering supplementary techniques (Lee & Yang, 2019). Although supplementary injection techniques are used, complete pulpal anesthetic success is challenging. The real challenge is when the tooth is a ‘hot tooth’ (Nusstein, Reader & Drum, 2010). Additional symptoms of mechanical allodynia (Lolignier, Eijkelkamp & Wood, 2015) and pulpal hyperemia also hinder the otherwise painless endodontic therapy.

The American Association of Endodontists divides pulpotomy/pulp amputation into necessary amputation and mortal/devitalization/mummification/amputation (Ehrmann, 1988). The mummification amputation includes mummification of pulp tissue with medicament and later removal of a devitalized pulpal tissue (Rahmawati, Nasserie & Adang, 2012). The devitalizing or mummification procedure can be a painless alternative to patients who experience hyperalgesia before or during endodontic therapy. This two-step technique not only mummifies the pulp tissue but also controls the hemorrhage and evades the administration of local anesthetic solution in a subsequent appointment (Rahmawati, Nasserie & Adang, 2012; Walimbe et al., 2015; Zhen-ya, 2013). Formaldehyde and its variants are routinely used devitalizing agents in pulpotomy procedures in primary molars for more than 100 years (Trask, 1972; Nunn, Smeaton & Gilroy, 1996; Peng et al., 2006). However, the worldwide concern over formaldehyde’s classification as carcinogenic in humans is primarily based on extrapolation from laboratory animal studies using very high doses of formocresol (Milnes, 2008a; Milnes, 2008b). An investigation done by Bagrizan et al. (2017) concluded that it is unlikely that the microgram quantities of formaldehyde utilized in the vital pulpotomy procedure could overwhelm the aforementioned biological pathways and escape into circulation.

To the best of the authors’ knowledge, there are no documented cases of systemic distribution of formocresol in humans. Hence, in the present investigation two pulpal medicaments containing different quantities of paraformaldehyde with lidocaine are used to compare its efficacy after the failure of IANB and supplemental intraligamentary injection in patients experiencing symptomatic irreversible pulpitis in the permanent mandibular tooth.

## Materials & Methods

### Study design

**Figure 1:**
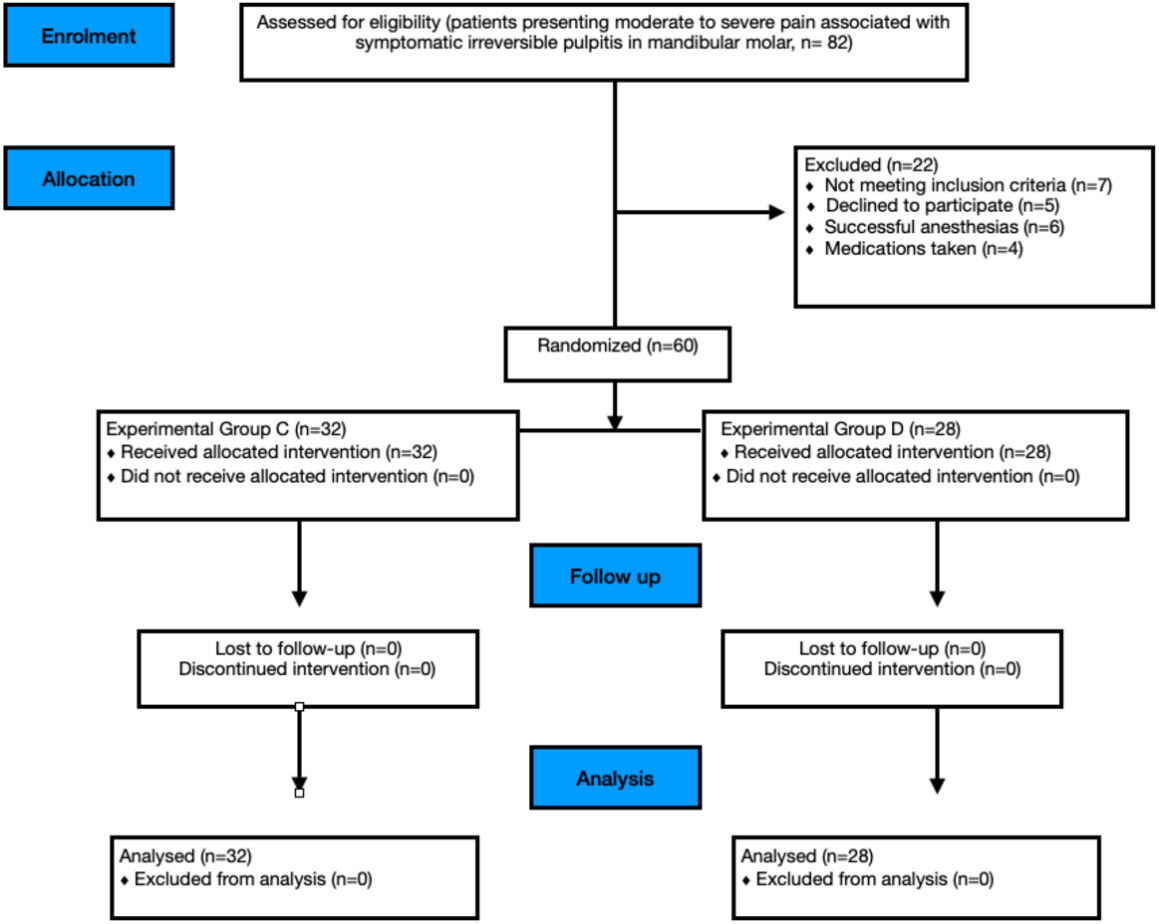
Study CONSORT flow diagram.

This study was designed as a prospective, two-arm, parallel-group, double-blind, randomized clinical trial that adhered to the Consolidated Standards of Reporting Trials (CONSORT) guidelines (Fig. 1). The research protocol was approved by the Institutional Ethics Committee of Institute of Medical Sciences (IMS) and SUM Hospital, Siksha ‘O’ Anusandhan (Deemed to be University) (DMR/IMS.SH/SOA/180042; date: 25th May 2018) and registered in the Clinical trials registry of India with no. CTRI/2018/07/014758.

The study was conducted in the Department of Conservative Dentistry, and Endodontics and patients were allocated into two groups with an allocation ratio of 1:1. All included patients (Table 1) signed a written informed consent after the nature of the study, its objectives, procedures, benefits and potential discomforts were fully explained and were given a copy of it. The patients aged 18 years and above were enrolled from the Department of Conservative Dentistry and Endodontics outpatient clinic from August 2018 till September 2019 as the sample size goal and the length of follow-up goal was reached.

**Table 1:** Eligibility criteria for patients to be included in the trial.

Inclusion criteria	
1.	Mandibular molar (first or second) with symptomatic irreversible pulpitis.
2.	Age—18 years and above.
3.	Medical status (Health condition according to American Society of Anesthesiologists class I or II).
4.	Tooth exhibiting a positive and exaggerated response to the electric pulp test (Denjoy DY310 Dental Pulp Tester, Denjoy Dental Co, Hunan, China) and cold pulp sensibility test with Endo-ice (Endo-Ice Spray; Henry Schein, Melville, NY )
5.	Patient experiencing moderate to severe pain even after administration of IANB
6.	Patient experiencing moderate to severe pain even after administration of intra-ligamentary LA administration.
7.	Tooth in question should not have any periapical radiolucency on intraoral peri-apical radiographs
Exclusion criteria	
1.	Medication that would alter pain perception
2.	Radiographic appearance of periapical pathology in the associated tooth
3.	Medical history that fall under compromised medical complexity status classification (MCS2 or MCS 3) or data collection (*e.g.*, facial paresthesia)
4.	Ongoing pain in more than one mandibular molar or attained anesthesia
5.	Analgesics taken within 12 h before endodontic treatment.

### Sample size estimation

With a type I error of 0.05 and statistical power of 80%, the minimum sample size was 60 participants to categorize a clinically significant difference of 30% in post-endodontic pain and bleeding incidence between the experimental groups. The number was escalated to 82 candidates participating in this investigation to account for exclusions and dropouts. Power and sample size calculation were estimated using PS software Version 3.1.2 (https://biostat.app.vumc.org/wiki/Main/PowerSampleSize).

### Diagnosis

**Figure 2:**
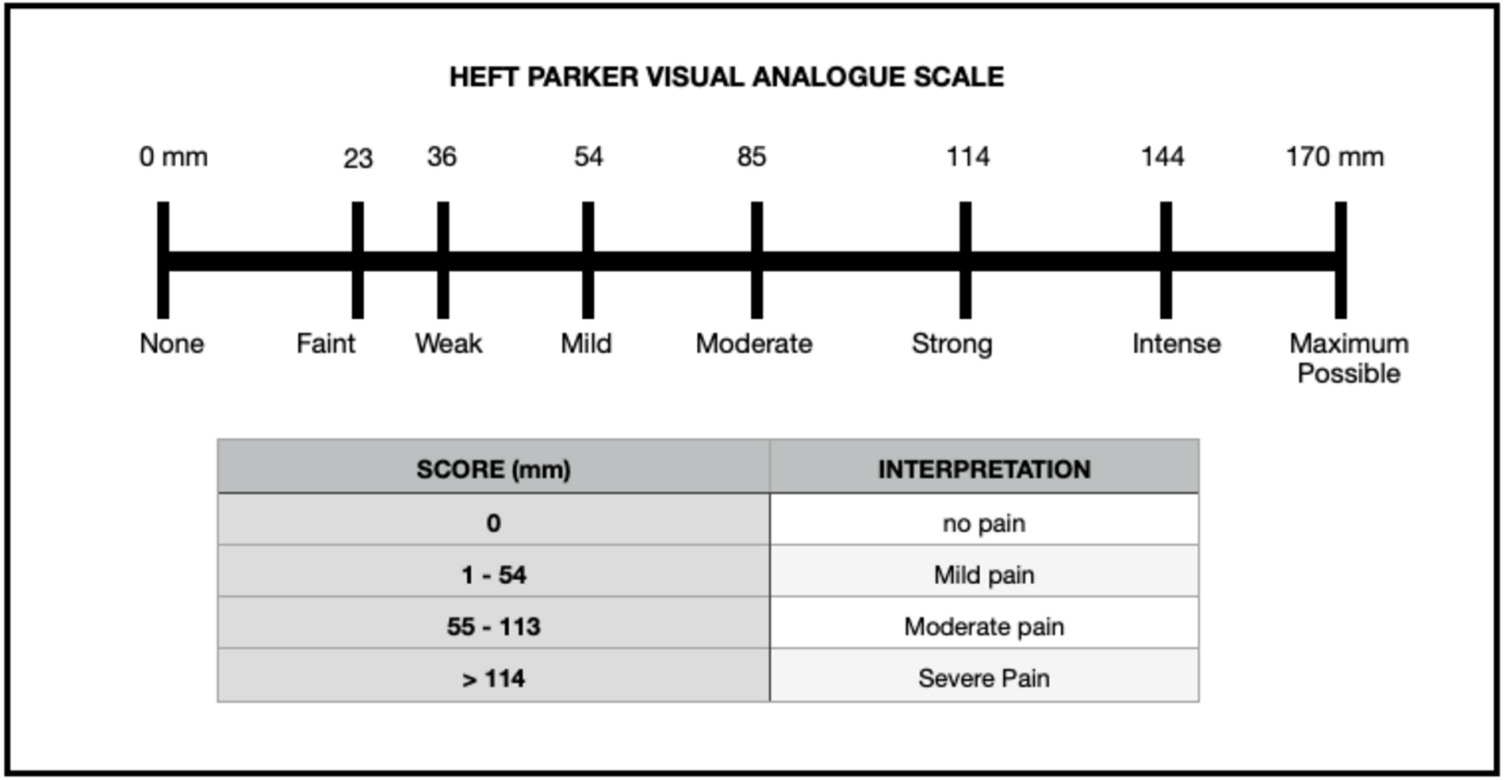
Heft–Parker visual analogue scale.

The diagnosis of symptomatic irreversible pulpitis was made based on the following: (a) patient’s chief complaint of severe pain that was sharp, piercing or shooting in nature, lingers even after the hot or cold stimulus and subsiding on intake of medications. (b) Patients giving a history of nocturnal pain and referred pain to the ear and with the clinical examination of deep caries and radiographic examination of radiolucency involving enamel, dentin and pulp with no peri-apical radiolucency. Pre-operative (PO > 114 mm) pain response of 82 patients was recorded using the VAS (Fig. 2) by the investigator (LM) using the electric pulp test and cold pulp sensibility test. The inclusion criteria for the trial were the following: mandibular molar with symptomatic irreversible pulpitis experiencing pain even after the administration of conventional IANB and supplemental intra-ligamentary technique which was recorded as positive and exaggerated response to the electric pulp test and cold pulp sensibility test, and tooth in question without having any periapical radiolucency on intraoral peri-apical radiographs. Endo-Ice Spray (Henry Schein, Melville, NY) was used to evaluate the pulp’s sensibility, and the Denjoy DY310 Dental Pulp Tester was used to test the pulp (Denjoy Dental Co, Hunan, China). The appearance of bleeding (very abundant cherry red blood ironless; Lejri, Douki & Kallel, 2019) during access cavity preparation indicated pulp hyperemia and pulpitis.

### Endodontic procedure

A standard IANB of 1.8ml was administered by investigator LM to 82 patients using 0.55 × 25 mm/24 × 1needle size, 2 ml syringe (DISPO VAN, HMD LTD) and 2% lidocaine with adrenaline (LOX2% Adrenaline 1: 200000, Neon, India). The access cavity preparation under a rubber dam was initiated after fifteen minutes of anaesthesia administration using Endo Access bur (Dentsply, Maillefer, Germany) and Endo-Z bur (Dentsply, Maillefer, Germany).

The operator RB blinded to the present study, continued endodontic treatment for patients who recorded no pain either to the IANB or IANB with intralingamentary injection. The adjuvant supplemental intraligamentary injection of 0.2 ml on each side of the tooth (buccal, lingual, mesial and distal) was administered with ligajet intraligamentary jet injector (Micro Mega) using 30 gauge needle and cartridge containing lignocaine hydrochloride 2% with adrenaline 1:80000 by the investigator LM. The needle was inserted in the periodontal ligament and advanced until resistance was felt. Only those patients who failed to obtain pulpal anesthesia and still experienced pain or discomfort during access cavity prepration, the case was transferred to the principal investigator (NS) and was included in the trial.

The patient was assigned randomly to one of two study groups: Group C (Caustinerf without arsenic, Septodont, France) or Group D (D-Pulp, Ammdent, India) using Excel software (Excel; Microsoft, Redmond, WA). Caustinerf by Septodont (Septodont, Lancaster, PA, USA) and D-Pulp by Ammdent contain lidocaine and paraformaldehyde in its composition. However, the quantities of paraformaldehyde in D-Pulp is 460 mg in 1gm and in Caustinerf it is 180 mg in 1 gm. In both groups, after recording the bleeding score or BS1 (0-no bleeding, 1-slight bleeding, 2-obvious bleeding), the minuscule quantity of Caustinerf or D-pulp was placed in the pulp chamber. Next, the tooth was sealed with the premixed thick zinc oxide-based temporary filling material (TEMPFILL-G, India). The patient was advised to take an analgesic medicament (Ketorol-DT, Dr Reddy’s pharmaceuticals, Hyderabad, India) if they experience pain within 24–48 h.

### Outcome

#### Pain assessment and bleeding score

Participants were recalled after 24–48 h (2nd visit) and asked about their pain status and intake of prescribed medication. Radiographic examinations with intraoral radiograph revealed similar findings in the periapical region as in first visit. Those patients who reported not taking painkillers after the first visit were sent to the second investigator, who was blinded to the investigation for further assessment. The second investigator (SB) was blinded to randomization, did rubber dam isolation of the involved tooth and removed the temporary restoration and pulpal medicament. After access opening and complete removal of the pulp in pulp chamber with an Endo-Z bur (Dentsply, Maillefer, North America), Access Opening (AO) visual analogue score was recorded.

A #10K file was introduced in each canal of the mandibular molar, and participants were asked to rate their pain once more using Heft Parker VAS which was recorded as canal instrumentation or CI. Also, the bleeding score of each canal was recorded as bleeding score 2 or BS2. Ability to enter the pulp chamber and instrument the root canal without pain (VAS rating of 0) or mild pain (VAS rating less than or equal to 54 mm) (Fig. 2) and comfortable completion of endodontic therapy without additional administration of local anaesthetic in the consecutive visit (2nd visit) determined the clinical success of the pulpal medicament.

In this study, pain assessment before the application of pulpal medicament was recorded at three-time points: pre-operative (PO), post local anaesthetic administration (LA), caries excavation (EX) and at two-time points following the application of pulpal medicament (after 24–48 h): during complete access opening (AO), and canal instrumentation (CI).

The bleeding score was recorded at two-time points: before applying pulpal medicament as bleeding score 1(BS1) and after applying pulpal medicament after 24–48 h as bleeding score 2 (BS2).

#### Recall protocol (clinical and radiographic assessment)

Follow-up appointments were scheduled for all the patients after six months of completion of root canal therapy. Radiographic examinations of all treated patients in the first visit, second visit and six months after completion of root canal therapy were performed by investigator NS using the parallel technique. The following equipment was used: intraoral radiograph films (Kodak Carestream X-ray Film E Speed, New York, USA), dental X-ray unit (Acteon X-Mind DC X-ray, manufactured by De Gotzen, notified body Eurofins, Torino, Italy), Automatic Dental X-ray Film Processor (Velopex, London, UK). Assessment of periapical status was achieved by a consensus panel of two pre-calibrated experienced endodontists. Treatment was contemplated successful based on the following features: the absence of periodontal ligament widening, internal resorption, external resorption, furcation radiolucency, or periapical radiolucency.

### Statistical analysis

For statistical analysis, these data were scrutinized, coded, and entered into IBM SPSS statistics 24.0 (SPSS South Asia PVT LTD., http://www.spss.co.in). The Shapiro Wilk test was used to check the normal distribution of quantitative data. The data was not normally distributed (*p* < 0.001) for all variables, so non-parametric tests were used. The qualitative parameters like bleeding were compared using the Chi-square test. The quantitative variables were compared using the Mann Whitney test between groups. The Wilcoxon sign rank test was used for the repeated measure of pain scores before and after the procedure. An alpha error was set to 5%, and a *p*-value of less than 0.05 was considered statistically significant.

## Results

Of 82 patients assessed for eligibility, 60 (25 females and 35 males) were randomized (Fig. 1). Age and gender distribution did not differ significantly across groups.

### Pain status (VAS)

Table 2 shows a noteworthy decline in severity of pain in the second visit scheduled at 24hrs to 48hrs in the two groups (*p* > 0.05). During postoperative complete access opening (AO) and canal instrumentation (CI), there was a significant decrease in the mean VAS score between Group C and Group D (Table 2).

**Table 2:** Pain score (mm) in the first and second visit.

Endodontic visits	Pain score (VAS) in mm	Group				‘*t*’ value	*p* value
		C (*n* = 32)		D (*n* = 28)			
		Mean	SD	Mean	SD		
1st Visit	PO	136.81	20.102	136.86	20.951	−0.008	0.993
	LA	51.34	22.115	51.43	18.052	−0.016	0.987
	EX	88.34	21.11	91.14	14.646	−0.588	0.559
**Pain score (mm) after endodontic treatment**							
2nd Visit	AO	5.13	11.17	8.54	15.233	−0.997	0.323
	CI	11.94	22.772	14.43	23.005	−0.421	0.676

**Notes.**

SD Standard deviation VAS Visual analogue score mm millimeter PO Pre-operative LA Local anesthesia EX Excavation AO Access opening CI Canal Instrumentation

Table 3 compares post-treatment pain score by teeth type within group C and group D. In group C, both VAS score during AO and CI have higher mean pain score for first molar teeth than the second molar teeth and were found to be significantly different (*p* < 0.05). Similarly, in group D, both AO and CI visual analogue scores have higher mean pain scores for first molar teeth than the second molar teeth. However, the difference was not statistically significant (*p* > 0.05).

**Table 3:** Comparison of post treatment pain score by teeth type within each group in 2nd visit.

Mandibular tooth type	Groups VAS SCORE in mm							
	C				D			
	AO		CI		AO		CI	
	Mean	SD	Mean	SD	Mean	SD	Mean	SD
Second molar	0	0	1.13	4.50	4.54	11.40	10.38	20.22
First molar	10.25	14.20	22.75	28.32	12.00	17.57	17.93	25.33
Mann Whitney ‘*p*’ value	0.008		0.011		0.222		0.237	

**Notes.**

SD Standard deviation VAS Visual analogue score mm millimeter AO Access opening CI Canal Instrumentation

In Table 4, the extent of deroofing or the extent of removal of the pulp chamber and placement of pulpal medicament. VAS score was statistically significant in the second visit for cases in which complete deroofing was possible. Complete deroofing resulted in VAS = 0 during AO (*p* − 0.243) and CI (*p* − 0.129) in group C. In Group D as well VAS = 0 during AO (0.288) and CI (0.141). In group C, before deroofing, mean VAS of 5.75 ± 10.64 and 17.50 ± 32.40 during the AO and CI procedure was recorded, respectively. In cases where partial deroofing was possible during AO, mean VAS of 7.87 mm ± 13.943 mm and 16.13 ± 22.25 during CI was recorded. There was no statistically significant result between before or partial deroofing in both groups.

**Table 4:** Postplacement of medicament pain score by extent of de-roofing (2nd visit).

Extent of de-roofing	Pain score in VAS (mm)							
	Group C				Group D			
	AO		CI		AO		CI	
	Mean	SD	Mean	SD	Mean	SD	Mean	SD
Before de-roofing	5.75	10.647	17.50	32.404	13.57	17.472	26.14	26.873
Partial de-roofing	7.87	13.943	16.13	22.251	9	16.1	13.81	23.019
Complete de-roofing	0	0	0	0	0	0	0	0
Kruskal Wallis ‘*p*’ value	0.243		0.129		0.288		0.141	

**Notes.**

SD Standard deviation VAS Visual analogue score mm millimeter AO Access opening CI Canal Instrumentation

### Bleeding score

There was a significant decrease in the overall bleeding score in both groups (*p* = 0.000). There was the presence of visible bleeding (96.9% of Group C patients and 96.4% of Group D patients) in the first visit (BS1). Post administration of pulpal medicament, there was a statistically significant reduction in bleeding from the pulp chamber in the second visit (BS2) in Group C,93.8% and Group D 89.3% had no bleeding (*p* = 0.533) (Table 5).

**Table 5:** Pre- and post-treatment bleeding score.

	Bleeding score	Group			
		C		D	
		No.	%	No.	%
BS1(1st visit)	Slight bleeding	1	3.10	1	3.60
	Obvious bleeding	31	96.90	27	96.40
BS2(2nd visit)	No bleeding	30	93.80	25	89.30
	Slight bleeding	2	6.30	3	10.70
	Obvious bleeding	0	0	0	0
* Marginal homogeneity test ‘*p*’ value		0.000		0.000	

**Notes.**

BS1 Bleeding score in 1st visit BS2 Bleeding score in 2nd visit

### Follow up visits (clinical assessment)

The recall rate was 100% for patients. At 6-month recall, all patients who received pulpal medicament were clinically asymptomatic.

### Follow up visit (radiographic assessment)

Two experienced, independent investigators evaluated the postoperative periapical radiographs. Kappa values for inter-examiner agreement on outcome diagnosis using periapical radiography (PA) was 0.86–0.92 (Table 6). All treated teeth with pulpal medicament showed favourable outcomes at 6-month follow-up.

**Table 6:** Kappa values for inter-examiner agreement on outcome diagnosis using periapical radiography (PA).

Follow up after six months of root canal completion	Inter-examiner agreement
PA	0.86–0.92

**Notes.**

0.40–0.60 moderate agreement, 0.60–0.70 substantial agreement, 0.80–1.0 almost total agreement.

## Discussion

Pain perception and intervention effectiveness are influenced by various psychological factors (De Matos et al., 2020). Hyperalgesia refers to an increased pain sensation than what one would experience typically after a noxious stimulus (Murray, 2009). Many individuals experiencing such pain opt for tooth extraction due to pain and have experienced a gradual escalation in pain responses following sensory stimulation, but tolerate these hyperalgesic responses and only seek care when the pain worsens (Henry et al., 2009). Possible reasons could be that there is altered sodium channel (NaCh) expression to acute pulpal pain mechanisms at single sites or significant NaCh accumulations in small-diameter myelinated fibers, where their activation could contribute to the sharp, shooting characteristics of spontaneous pulpal pain that originates from A-delta fibers (Henry et al., 2009). Such patients pose a challenge while performing root canal treatment. Additional local anaesthetic administration methods fail to work in cases where increased sensitization of nociceptors results from pulpal inflammation (Goodis, Poon & Hargreaves, 2006).

Several devices like Stabident, X tip, and Intraflow inject the local anesthetic solution into the cancellous bone adjacent to the tooth. However, these devices are technique sensitive and cause more discomfort to already stressed patients in inexperienced operators’ hands. These devices functioning depends mostly on locating and drilling the perforation sites, which might not be well accepted by patients. These devices are not only expensive but also have high maintenance costs. When not appropriately assembled, anesthetic solutions often leak from these devices (Saxena et al., 2013).

Alternative aesthetic solutions are available which are used for IANB for treatment of irreversible pulpitis. Mepivacaine, prilocaine and articaine are a few of such alternatives for lidocaine. However, a recent network meta-analysis revealed no difference in the efficacy of these alternatives, as mentioned above, when compared with lidocaine (Nagendrababu et al., 2019).

The simplistic approach of using pulpal medicament overcomes the problems associated with the failure of IANB in irreversible pulpitis cases. Nevertheless, several case reports and review papers have assessed its effects, yet the findings were conflicting, contradicting and controversial (Ballal, 2008; Bataineh, Al-Omari & Owais, 1997; Di Felice & Lombardi, 1998; Dumlu et al., 2007; Heling, Ram & Heling, 1977; Lee, Choi & Park, 2016; Milnes, 2008a; Milnes, 2008b; Özgöz et al., 2004; Özmeriç, 2002; Srivastava et al., 2011; Tortorici, Burruano & Difalco, 2007; Yalçın et al., 2003; Yavuz et al., 2008). These patients who complained of pain or bony changes speculated due to placement of these pulpal medicaments from one week (Lee, Choi & Park, 2016) to a maximum of three months’ time duration (Srivastava et al., 2011).

A standardized study protocol can aid in eliminating the confounding effects of intraoperative and postoperative variables on the use of pulpal medicaments (Ahmed et al., 2020). Therefore the present investigation was intended as a randomized, double-blind clinical trial with a 1:1 allocation ratio.

Randomization is a method of regulating and decreasing potential bias by maintaining an equilibrium of known and unidentified prognostic factors in the participants of the experimental groups while removing selection or allocation bias (Al-Rawhani, Gawdat & Wanees Amin, 2020). Blinding of both patients and investigators in this trial was done. This decreases performance and ascertainment bias (Al-Rawhani, Gawdat & Wanees Amin, 2020).

The present study utilized the Heft-Parker visual analogue scale. This visual analogue scale is a composite metric scale comprised of 6 category scale descriptors arranged unevenly on a horizontal line of 170-mm, offering patients several signals that may improve communication (Attar et al., 2008). Patients were educated on how to use the scale to improve consistency and eliminate bias. The current study revealed a significant reduction in postoperative pain after 24 to 48 h in both groups.

In this present trial, a head-to-head comparison was made to illustrate the pulpal medicaments’ efficacy. Two pulpal medicaments used in this study to control pain and bleeding in severely hyperalgesia patients with symptomatic irreversible pulpitis were Caustinerf by Septodont (Septodont, Lancaster, PA, USA) and D-Pulp by Ammdent. Both the pastes contain lidocaine and paraformaldehyde. However, the concentration of paraformaldehyde is more than twice in D-Pulp (460 mg in 1 gm) compared to Caustinerf (180 mg in 1 gm). Additionally, 1 gm of Caustinerf also contains two antiseptics: parachlorophenol (80 mg) and camphor(130 mg). The Caustinerf was clinically more effective than D-Pulp in reducing postoperative pain though statistically, there was no difference.

The pulpal medicament is most effective in complete de-roofing cases (Table 3). The reason could be that paraformaldehyde chemical binds with pulp cell protein, especially those amino acids that have dual peptide groups, primarily between the residues of the essential amino acid lysine, which prevent tissue autolysis. An assumption that the formation of methylene bridges between peptide groups of adjacent amino acids connects the protein molecules without changing their basic overall structure is probably the reason for increased tissue hardness and the altered chemical reactivity. This tissue fixative property of pulpal medicament was helpful in patients with evident pulpal hyperemia (Berger, 1972). In the first appointment (recorded as Bleeding Score 1 or BS1), after the placement of pulpal medicament, had controlled or no pulpal hyperemia (BS2) in both the intervention groups (Table 4). The pulpal medicaments made the pulp chamber and canal clear and visible for instrumentation. The paraformaldehyde alters blood flow and induces thrombus formation, leading to bleeding cessation (Berger, 1972; Chandrashekhar & Shashidhar, 2014).

The pain score was slightly higher in the mandibular first molar than in the second molar. It could be because, in the Indian population, the most common root canal morphology encountered in the second mandibular is two mesial canals and one canal in the distal aspect (Neelakantan et al., 2010). In contrast, the first mandibular molar has a higher incidence of four canals, two mesial canals and two distal canals (Chourasia et al., 2012). To achieve the full effect of devitalizes, probably the first molar requires slightly more quantity of paraformaldehyde based pulpal medicament than the second molars.

On the contrary to postoperative pain, there was no significant difference in the bleeding status post-operatively in mandibular first and second molar as both the mandibular molars have similar average pulp volume (Fanibunda, 1986). This study is in accordance with previous literature published that states that a contact time of paraformaldehyde or formaldehyde to pulp tissue for 24–48 h did not cause any significant alteration to the periapical tissue, which is seen in the present study as well (Berger, 1972; Chandrashekhar & Shashidhar, 2014; Walimbe et al., 2015).

From the results obtained, both the groups showed a significant reduction in pain and bleeding status 24 to 48 h after placing a minuscule quantity of pulpal medicament. Caustinerf has less concentration of paraformaldehyde, and pain control was either equal or more than D-pulp, clinically. Therefore, clinicians can prefer the use of Caustinerf over D-pulp. It can be concluded from the above trial that judicious use of paraformaldehyde containing pulpal medicament can reduce pain and pulpal hyperaemia during a subsequent endodontic procedure, especially in mandibular molars with a clinical diagnosis of irreversible pulpitis with cold hyperalgesia. This short duration of pulpal medicament does not result in any radiographic periapical changes.

### Study strengths and limitations

The present investigation is, to our knowledge, the only double-blind, randomized trial of this intervention in severely symptomatic irreversible pulpitis. The relatively small sample size is a limitation of this study. Secondly, the use of devitalizes is considered for endodontic pain management protocol, and formaldehyde-based products are not in use in many countries. However, the present study suggests the use of it can be an alternative, painless method of achieving painless endodontic therapy. However, it cannot negate that any potential subsequent more extensive randomized controlled trials should use more patient-centred endpoints such as increased follow up periods and additional diagnostic aids like CBCT to evaluate any periapical changes.

## Conclusion

Within the limitations of the present study, it can be concluded that both medicaments (Caustinerf and D-Pulp) exhibit comparable effectiveness in postoperative pain reduction in the treatment of irreversible pulpitis in mandibular molars.

## Supplemental Information

10.7717/peerj.13397/supp-1 Supplemental Information 1 Consort checklist

10.7717/peerj.13397/supp-2 Supplemental Information 2 Raw Data

## Additional Information and Declarations

### Data Availability

The following information was supplied regarding data availability: The raw data are available in the Supplemental File.

### Clinical Trial Registration

The following information was supplied regarding Clinical Trial registration:
CTRI/2018/07/014758.
